# Supplementary material for: Neural signatures of model-based and model-free reinforcement learning across prefrontal cortex and striatum
Source: eLife. 2026 Jun 22;14:RP106032. doi: 10.7554/eLife.106032 (PMC13286570; doi:10.7554/eLife.106032)
Supplement: Peer review history

# Editor report: eLife Assessment

**DOI:** 10.7554/eLife.106032.3.sa0

**Authors:** Thorsten Kahnt

**Affiliations:** National Institute on Drug Abuse Intramural Research Program United States

**Keywords:** Convincing, Important

This **important** study presents single-unit activity collected during model-based (MB) and model-free (MF) reinforcement learning in non-human primates. The dataset was carefully collected, and the statistical analyses, including the modeling, are rigorous. The evidence **convincingly** supports different roles for particular cortical and subcortical areas in representing key variables during reinforcement learning.

# Referee report: Reviewer #1 (Public review)

**DOI:** 10.7554/eLife.106032.3.sa1

Summary:

Using single-unit recording in 4 regions of non-human primate brains, the authors tested whether these regions encode computational variables related to model-based and model-free reinforcement learning strategies. While some of the variables seem to be encoded by all regions, there is clear evidence for stronger encoding of model-based information in anterior cingulate cortex and caudate.

Strengths:

The analyses are thorough, the writing is clear, the work is well-motivated by prior theory and empirical studies.

Weaknesses:

The authors have adequately addressed my prior comments.

# Referee report: Reviewer #2 (Public review)

**DOI:** 10.7554/eLife.106032.3.sa2

Summary:

The authors investigate single-neuron activity in rhesus macaques during model-based (MB) and model-free (MF) reinforcement learning (RL). Using a well-established two-step choice task, they analyze neural correlates of MB and MF learning across four brain regions: the anterior cingulate cortex (ACC), dorsolateral PFC (DLPFC), caudate, and putamen. The study provides strong evidence that these regions encode distinct RL-related signals, with ACC playing a dominant role in MB learning and caudate updating value representations after rare transitions. The authors apply rigorous statistical analyses to characterize neural encoding at both population and single-neuron levels.

Strengths:

(1) The research fills a gap in the literature, which has been limited in directly dissociating MB vs. MF learning at the single unit level and across brain areas known to be involved in reinforcement learning. This study advances our understanding of how different brain regions are involved in RL computations.

(2) The study used a two-step choice task Miranda et al., (2020), which was previously established for distinguishing MB and MF reinforcement learning strategies.

(3) The use of multiple brain regions (ACC, DLPFC, caudate, and putamen) in the study enabled comparisons across cortical and subcortical structures.

(4) The study used multiple GLMs, population-level encoding analyses, and decoding approaches. With each analysis, they conducted the appropriate controls for multiple comparisons and described their methods clearly.

(5) They implemented control regressors to account for neural drift and temporal autocorrelation.

(6) The authors showed evidence for three main findings:

(a) ACC as the strongest encoder of MB variables from the four areas, which emphasizes its role in tracking transition structures and reward-based learning. The ACC also showed sustained representation of feedback that went into the next trial.

(b) ACC was the only area to represent both MB and MF value representations.

(c) The caudate selectively updates value representations when rare transitions occur, supporting its role in MB updating.

(7) The findings support the idea that MB and MF reinforcement learning operate in parallel rather than strictly competing.

(8) The paper also discusses how MB computations could be an extension of sophisticated MF strategies.

Weaknesses:

(1) There is limited evidence for a causal relationship between neural activity and behavior. The authors cite previous lesion studies, but causality between neural encoding in ACC, caudate, and putamen and behavioral reliance on MB or MF learning is not established.

(2) There is a heavy emphasis on ACC versus other areas, but is unclear how much of this signal drives behavior relative to the caudate.

(3) The authors mention the monkeys were overtrained before recording, which might have led to a bias in MB versus MF strategy.

(4) The authors have responded to the weaknesses appropriately in the manuscript.

# Author comment: Author response

**DOI:** 10.7554/eLife.106032.3.sa3

**Authors:** Bruno Miranda, James L Butler, WM Nishantha Malalasekera, Timothy E Behrens, Peter Dayan, Steven Wayne Kennerley

**Affiliations:** Instituto de Medicina Molecular, Faculdade de Medicina, Universidade de Lisboa Lisbon Portugal; University of Oxford Oxford United Kingdom; University College London London United Kingdom; University of Oxford Oxford United Kingdom; Max Planck Institute for Biological Cybernetics Tuebingen Germany; University College London London United Kingdom

The following is the authors’ response to the original reviews.

**Public Reviews:**

**Reviewer #1 (Public review):**

Summary:

Using single-unit recording in 4 regions of non-human primate brains, the authors tested whether these regions encode computational variables related to model-based and model-free reinforcement learning strategies. While some of the variables seem to be encoded by all regions, there is clear evidence for stronger encoding of model-based information in the anterior cingulate cortex and caudate.

Strengths:

The analyses are thorough, the writing is clear, and the work is well-motivated by prior theory and empirical studies.

Weaknesses:

My comments here are quite minor.

The correlation between transition and reward coefficients is interesting, but I'm a little worried that this might be an artifact. I suspect that reward probability is higher after common transitions, due to the fact that animals are choosing actions they think will lead to higher reward. This suggests that the coefficients might be inevitably correlated by virtue of the task design and the fact that all regions are sensitive to reward. Can the authors rule out this possibility (e.g., by simulation)?

We fully agree with the reviewer that the task design has in-built correlations between transition and reward, and thus the correlation between neural selectivity for feedback and transition (Figure 3E) may be due to the different reward expectation after common or rare transitions. We did try to make this point in the manuscript:

This suggests that the brain treats being diverted away from your current objective equivalent to losing reward, which is sensible as the subject would normally expect lower rewards on rare trials if their reward-seeking behaviour was efficient.

We’ve now updated the wording of this statement to try and better make this point and avoid confusion that any non-reward-related encoding is involved:

“As the reward expectation will be higher on common compared to rare trials, this demonstrates that the brain encodes being diverted to an area with a lower reward expectation equivalent to actually receiving a low reward (and vice versa).”

We have also adjusted the significance test of this correlation to use a circular permutation test that accounts for correlations between the regressors. This test still found there to be significant correlation in all areas.

We have described this new permutation test in Methods:

“For comparing correlations between weights for different features (i.e., between transition and reward coding, Figure 3E), the null distribution of correlations observed in circularly shifted data was compared to the correlation seen in the actual data. This accounts for any correlations between features that existed in the task by preserving the structure of the design matrices.”

And updated the text in Results accordingly:

“All regions, but particularly ACC, encoded a common transition (at the time of transition) similar to a high reward (at the time of feedback), as there was a positive correlation between the coefficients for reward and transition (the transition parameter was signed such that common and rare transitions were equivalent to high and low rewards, respectively) (ACC r=0.4963, DLPFC r=0.3273, caudate r=0.4712, putamen, r=0.5052; all *p*<0.002 except DLPFC where p=0.006, circular permutation test; Figure 3E, S5).”

The explore/exploit section seems somewhat randomly tacked on. Is this really relevant? If yes, then I think it needs to be integrated more coherently.

We thank the reviewer for this comment. We agree that the motivation for the explore/exploit analysis was not sufficiently clear in the original version.

Our aim was not to introduce this as a separate or tangential effect, but rather to highlight how the task’s reward structure (with outcome levels stable for 5–9 trials) naturally created alternating periods favoring exploitation of a known high-value option versus exploration when outcomes changed. This feature of the task is tightly linked to MB-RL computations, as it requires integration of state-transition knowledge and updating across trials.

Importantly, we show previously in the manuscript that ACC encoded state-transition structure (i.e., common versus rare transition) and MB-value estimates (at choice epoch). However, here we aimed to highlight that the same region also modulated choice encoding as a function of whether the subject was in an exploratory or exploitative regime – by knowing another feature of the task that relies on state-transition and outcome. We have revised this section to better integrate it into the main logic of the paper:

“In our task, the outcome level (high, medium, low) of each second-stage stimulus remained the same for 5-9 trials before potentially changing. This design naturally created periods where subjects could ‘exploit’ the same Choice 1 to maximize reward for several trials; and other periods where they had to ‘explore’ different second-stage stimuli to optimize reward (as contingencies shifted). In classical MB-RL, the transition between reward states can be learned by keeping counts of observed transitions from a current state-action pair to a subsequent state, yielding a maximum-likelihood estimate of the environment’s dynamics [42]. In fact, knowledge about the reward contingency schedule could support decision-making in both exploitation – by enabling efficient choice when rewards are stable; and exploration – by guiding alternative behaviour most likely to yield improved outcomes (this is different from MF learning, where exploration is more random since the agent lacks explicit state-transition knowledge).

We thus repeated our decoding analysis of choice 1 stimulus identity, but this time limited trials to those where they had not received a high reward for the previous two trials (‘explore’ trials), and those where the previous two rewards had been the highest level (‘exploit’ trials). All regions encoded choice 1 for some duration of the choice epoch for both explore (p<0.002 in all cases, permutation test; Figure 7A) and exploit (p<0.002 in all cases; Figure 7B) conditions, but decoding accuracy was strongest in ACC. Choice 1 was less strongly decoded – particularly in ACC – in the former condition compared to the latter (p<0.002 for at least 140 ms in all cases, permutation test on differences observed; Figure 7C); and, also during exploitation, the ACC encoded choice 1 before the choice was even presented to the subject (Figure S8). This pre-choice ACC encoding in exploit trials may reflect the need to allocate cognitive (or attentive) resources to features – i.e., choice 1 stimulus identity – that are most certain predictors of important outcomes. As a control, we also decoded the direction of the Choice 1 (where choice was indicated via joystick movement), which was randomised each trial and therefore orthogonal to the stimulus that was chosen. Again, all four regions encoded its direction in both explore (p<0.002 in all cases; Figure 7D) and exploit (p<0.002 in all cases; Figure 7E). However, there were minimal differences in the strength of the representation between explore and exploit conditions (ACC, p=0.088, cluster-based permutation test; DLPFC p=0.016; caudate p=0.32; putamen p=1; Figure 7F). Therefore, exploit behaviour specifically upregulated relevant task parameters that were worth remembering across trials.”

**Reviewer #2 (Public review):**

Summary:

The authors investigate single-neuron activity in rhesus macaques during model-based (MB) and model-free (MF) reinforcement learning (RL). Using a well-established two-step choice task, they analyze neural correlates of MB and MF learning across four brain regions: the anterior cingulate cortex (ACC), dorsolateral PFC (DLPFC), caudate, and putamen. The study provides strong evidence that these regions encode distinct RL-related signals, with ACC playing a dominant role in MB learning and caudate updating value representations after rare transitions. The authors apply rigorous statistical analyses to characterize neural encoding at both population and single-neuron levels.

Strengths:

(1) The research fills a gap in the literature, which has been limited in directly dissociating MB vs. MF learning at the single unit level and across brain areas known to be involved in reinforcement learning. This study advances our understanding of how different brain regions are involved in RL computations.

(2) The study used a two-step choice task Miranda et al., (2020), which was previously established for distinguishing MB and MF reinforcement learning strategies.

(3) The use of multiple brain regions (ACC, DLPFC, caudate, and putamen) in the study enabled comparisons across cortical and subcortical structures.

(4) The study used multiple GLMs, population-level encoding analyses, and decoding approaches. With each analysis, they conducted the appropriate controls for multiple comparisons and described their methods clearly.

(5) They implemented control regressors to account for neural drift and temporal autocorrelation.

(6) The authors showed evidence for three main findings:

(a) ACC as the strongest encoder of MB variables from the four areas, which emphasizes its role in tracking transition structures and reward-based learning. The ACC also showed sustained representation of feedback that went into the next trial. (b) ACC was the only area to represent both MB and MF value representations.

(c) The caudate selectively updates value representations when rare transitions occur, supporting its role in MB updating.

(7) The findings support the idea that MB and MF reinforcement learning operate in parallel rather than strictly competing.

(8) The paper also discusses how MB computations could be an extension of sophisticated MF strategies.

Weaknesses:

(1) There is limited evidence for a causal relationship between neural activity and behavior. The authors cite previous lesion studies, but causality between neural encoding in ACC, caudate, and putamen and behavioral reliance on MB or MF learning is not established.

We agree with the reviewer that the present study does not establish causal relationships, and we do not claim otherwise in the manuscript. Our work was designed as a comprehensive characterization of neural activity across ACC, DLPFC, caudate, and putamen during reward-seeking decision-making. By systematically comparing MB- and MF- RL signals across these regions, we provide new insights into the division of labor and cooperative interactions within cortico-striatal networks.

While causal manipulations (e.g., lesions, inactivations, stimulation) are indeed required to directly establish necessity or sufficiency, correlational studies such as ours play a crucial role in identifying where and how computationally relevant signals are represented. Importantly, our findings align with and extend prior causal work, for example showing that ACC and striatal lesions disrupt MB control. Thus, our study contributes a detailed functional mapping of MB and MF RL encoding across multiple nodes of this circuit, which serves as an important foundation for future causal investigations (e.g., using transcranial ultrasound stimulation).

(2) There is a heavy emphasis on ACC versus other areas, but it is unclear how much of this signal drives behavior relative to the caudate.

We appreciate the reviewer's observation regarding this matter. Our intention was not to place a heavy emphasis on ACC, rather this came naturally from the data. The ACC demonstrated considerably more robust and enduring neural activity compared to other brain regions – for instance, reward-related signals in the ACC continued well beyond individual trials (Fig. 2A-B), and encoding of state transitions remained active from the initial transition through to the feedback phase (Fig. 3A-B). By comparison, distinctions among other regions were less pronounced, which naturally resulted in the ACC receiving greater attention in our analytical findings.

We acknowledge that the caudate plays an essential and complementary role in driving behavior, and we believe that this is emphasized in the two key subsections of our “Results”. First, caudate neurons encoded model-based choice values (Fig. 4A, 4C) and uniquely remapped these values following rare transitions (Fig. 5), reflecting flexible adjustment of action values. Second, decoding analyses showed that both ACC and caudate populations predicted first-stage choices (Fig. 6C), linking their activity directly to behavioral decisions. In the Discussion section, we also highlight that “the distinctive caudate signal of updating (flipping) the value estimates of the currently experienced option on rare trials” goes beyond a “general temporal-difference RPE” and rather supports “the role of caudate in MB valuation”.

(3) The role of the putamen is somewhat underexplored here.

Our analyses were conducted in an identical manner across all four recorded regions (ACC, DLPFC, caudate, and putamen), and we consistently reported the results for putamen alongside the others. For example, in the Results section we describe how “both caudate and putamen encoded the reward from the previous trial negatively during the feedback period of the current trial” (Fig. 2F-G), and that “all regions had a significant population of neurons that encoded MB-, but not MF-, derived value” including putamen (Fig. 4F). Similarly, we show that putamen, like caudate, encoded a dopamine-like RPE signal at feedback (“both caudate and putamen neurons clearly responded at feedback with the parametric features of a dopamine-like RPE”; Discussion). These findings align with previous work linking the putamen to MF learning and are discussed explicitly in the context of MF-MB dissociations. We therefore believe that the putamen was not underexplored, but rather that its contribution was more circumscribed relative to ACC and caudate because the signals observed were quantitatively weaker and less distinctive for MB computations.

(4) The authors mention the monkeys were overtrained before recording, which might have led to a bias in the MB versus MF strategy.

We agree that extensive training can influence the balance between MB and MF in choice behaviour and neuronal responses.

In a previous comprehensive behavioral analysis of the same dataset (Miranda et al., 2020, PLoS Computational Biology - ref. 36, Figure S6B) we showed that both MB and MF strategies contributed to behavior, with MB dominance stable across weeks of testing – supporting that overtraining did not eliminate MF influences (but rather stabilized a mixed strategy with robust MB contributions).

In the same manuscript, we have also: (i) cautioned the readers when comparing our results to data from the original human studies; (ii) acknowledged that our extensive training cannot address earlier phases of learning in which sensitivity to the task structure is first acquired; and (iii) also provided task-related reasons for such MB dominance – as training made the transition structure well learned (making MB computationally less costly and faster to implement) and the non-stationary outcomes favored the flexibility of MB strategies.

In the present manuscript, we also have acknowledged that overtraining may have shifted neural signals toward stronger MB representations, or alternatively enabled more sophisticated task representations:

“On the other hand, MF-based estimates were neither as striking nor as specific to striatal regions as expected and observed in previous studies [18]. The monkeys were extensively trained on the task before recordings commenced, which may have caused a shift towards both MB behaviour and MB value representation within the striatum. Alternatively, this training may have allowed more sophisticated representations to occur, such as using latent states to expand the task space [54].”

Importantly, we strongly believe that this possibility does not detract from our main finding that both MB and MF signals were present across regions, with ACC showing the strongest multiplexing of the two.

(5) The GLM3 model combines MB and MF value estimates but does not clearly mention how hyperparameters were optimized to prevent overfitting. While the hybrid model explains behavior well, it does not clarify whether MB/MF weighting changes dynamically over time.

We appreciate this comment and would like to note that, for completeness, we have on several occasions directed the reader to our prior behavioural analysis of the same dataset (Miranda et al., 2020, PLoS Computational Biology, ref 36). In that work, we provide a full and detailed description of both the task and the computational modeling approach (see particularly the “Model fitting procedures” section). Furthermore, our model-fitting was grounded in the MF/MB RL framework used in the original human two-step study (Daw et al., 2011); and the fitting procedures also followed previous studies (Huys et al., 2011).

Hyperparameters – including the MB/MF weighting parameter (ω) - were estimated using maximum likelihood under two complementary approaches and with priors providing regularization across sessions. First, we performed a fixed-effects analysis, in which parameters were estimated independently for each session by maximizing the likelihood separately; secondly, we conducted a mixed-effects analysis, treating parameters as random effects across sessions within each subject. The effect of the prior procedure reduces the risk of overfitting by constraining parameters based on their empirical distributions, rather than allowing unconstrained session-by-session estimates. Finally, all model fitting procedures were verified on surrogate generated data.

With regard to dynamic weighting, our approach – consistent with most two-step studies – assumed ω to be constant across trials within each session. This was a deliberate choice, both for comparability with prior work and because our subjects were extensively trained, making session-level stability of strategy weights a reasonable assumption. Indeed, our analyses showed no systematic drift in ω across sessions, suggesting that MB/MF balance was stable over sessions. While approaches that allow dynamic ω estimation are possible, we believe such extensions would likely have minimal impact in the current dataset.

(6) It was unclear from the task description whether the images used changed periodically or how the transition effect (e.g., in Figure 3) could be disambiguated from a visual response to the pair of cues.

All images were kept constant across sessions. Common/Rare transitions themselves were not explicitly cued, but rather each second-stage state was associated with a specific background colour, followed ~1s later by the presentation of two specific second-stage choice cues (Figure 1B). Hence the subject could infer whether they were transitioned down a Rare or Common path by the background colour, which can be disambiguated in time from the visual responses to the second-stage cues. We’ve updated the Results text to make this clearer:

“Tracking the state-transition structure of the task is imperative for solving the task as a MB-learner. All four regions encoded whether the current trial’s first-stage choice transitioned to the common or rare second-stage state (which could be inferred by a change in background colour immediately after choice indicating which second stage state they had just entered, Figure 1A).”

**Recommendations for the authors:**

**Reviewer #1 (Recommendations for the authors):**

(1) Figure 7 appears to be missing.

We thank the reviewer for pointing this out. Figure 7 was inadvertently omitted in the previous version and has now been included in the revised manuscript.

(2) No stats reported in the section on explore/exploit.

We apologise for this oversight. This section now also reports the relevant statistics:

“We thus repeated our decoding analysis of choice 1 stimulus identity, but this time limited trials to those where they had not received a high reward for the previous two trials (‘explore’ trials), and those where the previous two rewards had been the highest level (‘exploit’ trials). All regions encoded choice 1 for some duration of the choice epoch for both explore (p<0.002 in all cases, permutation test; Figure 7A) and exploit (p<0.002 in all cases; Figure 7B) conditions, but decoding accuracy was strongest in ACC. Choice 1 was less strongly decoded – particularly in ACC – in the former condition compared to the latter (p<0.002 for at least 140 ms in all cases, permutation test on differences observed; Figure 7C); and, also during exploitation, the ACC encoded choice 1 before the choice was even presented to the subject (Figure S8). This pre-choice ACC encoding in exploit trials may reflect the need to allocate cognitive (or attentive) resources to features – i.e., choice 1 stimulus identity – that are most certain predictors of important outcomes. As a control, we also decoded the direction of the Choice 1 (where choice was indicated via joystick movement), which was randomised each trial and therefore orthogonal to the stimulus that was chosen. Again, all four regions encoded its direction in both explore (p<0.002 in all cases; Figure 7D) and exploit (p<0.002 in all cases; Figure 7E). However, there were minimal differences in the strength of the representation between explore and exploit conditions (ACC, p=0.088, cluster-based permutation test; DLPFC p=0.016; caudate p=0.32; putamen p=1; Figure 7F).”

(3) Make sure that error bars are explained in all figure captions where appropriate.

We apologise that this information was absent. Error bars always represent the standard error of the mean. This has now been added to all relevant figure legends.

**Reviewer #2 (Recommendations for the authors):**

Overall, I think this is a great manuscript and was presented clearly and succinctly. I have some minor suggestions:

(1) Typo: Abstract "ACC, DLPFC, caudate and striatum" I think should be "caudate and putamen".

We have amended this incorrect reference in the introduction:

“One such task that does enable the dissociation of MB and MF computations is Daw et al. (2011)’s ‘two-step’ task [18]. It contains a probabilistic transition between task states to uncouple MF learners (who would assign credit to which state was rewarded regardless of the transition) from MB learners (who would appropriately assign credit based on the reward and transition that occurred). Rodents [19], monkeys [36], and humans [18] all use MB-like behaviour to solve the task. Evidence in rodents suggests dorsal anterior cingulate cortex (ACC) tracks rewards, states, and the probabilistic transition structure, and that ACC is essential in implementing a MB-strategy [37]. Here, we compare primate single neuron activity of 4 different subregions implicated in reward-based learning and choice (ACC, dorsolateral PFC (DLPFC), caudate, and putamen) during performance of the classic two-step task, and demonstrate signatures of MB-RL primarily in ACC, and MF-RL signatures most notably in putamen.”

(2) Could the authors provide a rationale for why they did the single-level encoding the way they did, instead of running an ANOVA?

We thank the reviewer for this point. We are not entirely certain which specific ANOVA approach is being suggested, but our rationale for using a GLM-based encoding analysis is that such approach allows us to model continuous, trial-by-trial variables (e.g., value signals, prediction errors, transitions) while simultaneously controlling for multiple correlated predictors. This approach is widely used in systems neuroscience (particularly in decision-making research) offering analytical flexibility and comparability with prior approaches.

(3) How were the 20 iterations for decoding decided? That seems low.

We do not agree that 20 repetitions of 5-fold cross validation is low. The error bars in panels 6C-E demonstrate what low variance occurred across these 20 repetitions. It is the average of these low variance repetitions against which we performed statistics by performing a permutation test where these 20 repetitions were repeated a further 500 times.

(4) It was unclear to me how the authors reached the conclusion "Thus, caudate activity appeared to represent the value of the state the subject was currently in." when the state value wasn't computed directly. I don't see how encoding the chosen and unchosen option is the same as the state the animal is in, which should also incorporate where the animal is in a block of trials or session, and the knowledge regarding the chosen and unchosen option.

We agree with this point and have tempered this statement:

“Thus, caudate’s encoding of an option’s value also reflected the availability of the option.”

(5) Figures 1C, D, and E were not legible to me even at 200% zoom.

We apologise for this oversight. We’ve now updated panels 1C-E to a more readable size:

(6) There is a Figure 2H in the figure legend, but the panel appears to be missing from Figure 2.

This text has been removed.

(7) Figure 2: It would've been nice to see F and G for all areas.

We have now added this data as additional panels in Figure 2.

(8) Figure 3: How is the transition disambiguated from a visual response to the set of images?

This was indicated by the background changing colour to that of the learned second stage state before the actual choices were presented. We’ve updated the Results text to make this clearer:

“Tracking the state-transition structure of the task is imperative for solving the task as a MB-learner. All four regions encoded whether the current trial’s first-stage choice transitioned to the common or rare second-stage state (which was indicated by a change in background colour before the second stage choices were presented, Figure 1A).”

(9) Figure 4F: Is this collapsed across time points? So neurons that were significant at any time? I'm confused how Figure 4A relates to 4F, as 4A shows much lower percentages of significant neurons.

Figure 4F counts the total number of neurons that had a significant period of encoding at any timepoint over the epoch (as assessed with a length-based permutation test). Whereas, 4A shows the amount of significant encoding neurons at any one time point. Investigating this further, we found that the encoding was dynamic with different neurons encoding different parts of the epoch. We have now added a new supplementary figure to highlight this and refer to it in Results:

“Examination of the strongest signal observed, ACC’s encoding of MB Q-values, showed a dynamic pattern with different neurons encoding the signal at different parts of the epoch (Figure S6). When aggregating the number of significant coders throughout the epoch, and examining the specificity of MB versus MF coding, we found that all regions had a significant population of neurons that encoded MB-, but not MF-, derived value (30, 18.72, 23 and 24% of neurons in ACC, DLPFC, caudate and putamen respectively; all p<0.0014 binomial test against 10% (as the strongest response to either of the two options was used); Figure 4F).“

(10) Data/ code could be made publicly available instead of upon request.

All data and code to reproduce figures are now available at https://github.com/jamesbutler01/TwoStepExperiment. The manuscript has been updated to reflect this:

Data and materials availability:

All data and code to reproduce figures are available at https://github.com/jamesbutler01/TwoStepExperiment.
